# Use of bioreactors for culturing human retinal organoids improves photoreceptor yields

**DOI:** 10.1186/s13287-018-0907-0

**Published:** 2018-06-13

**Authors:** Patrick Ovando-Roche, Emma L. West, Matthew J. Branch, Robert D. Sampson, Milan Fernando, Peter Munro, Anastasios Georgiadis, Matteo Rizzi, Magdalena Kloc, Arifa Naeem, Joana Ribeiro, Alexander J. Smith, Anai Gonzalez-Cordero, Robin R. Ali

**Affiliations:** 1 Department of Genetics, UCL Institute of Ophthalmology, 11–43 Bath Street, London, EC1V 9EL UK; 2 NIHR Biomedical Research Centre at Moorfields Eye Hospital NHS Foundation Trust and UCL Institute of Ophthalmology, City Road, London, EC1V 2PD UK

**Keywords:** Pluripotent stem cells, Retinal organoids, Bioreactors, Photoreceptors

## Abstract

**Background:**

The use of human pluripotent stem cell-derived retinal cells for cell therapy strategies and disease modelling relies on the ability to obtain healthy and organised retinal tissue in sufficient quantities. Generating such tissue is a lengthy process, often taking over 6 months of cell culture, and current approaches do not always generate large quantities of the major retinal cell types required.

**Methods:**

We adapted our previously described differentiation protocol to investigate the use of stirred-tank bioreactors. We used immunohistochemistry, flow cytometry and electron microscopy to characterise retinal organoids grown in standard and bioreactor culture conditions.

**Results:**

Our analysis revealed that the use of bioreactors results in improved laminar stratification as well as an increase in the yield of photoreceptor cells bearing cilia and nascent outer-segment-like structures.

**Conclusions:**

Bioreactors represent a promising platform for scaling up the manufacture of retinal cells for use in disease modelling, drug screening and cell transplantation studies.

**Electronic supplementary material:**

The online version of this article (10.1186/s13287-018-0907-0) contains supplementary material, which is available to authorized users.

## Background

Visual impairment caused by inherited retinal degenerations as well as more complex heterogeneous retinal diseases such as age-related macular degeneration (AMD) and glaucoma are mainly due to the dysfunction or loss of key retinal cells such as retinal pigment epithelium (RPE) cells, photoreceptors or retinal ganglion cells (RGCs). Whilst animal models have provided many important insights into retinal disorders, they often do not recapitulate key aspects of human pathophysiology and, as a result, the molecular mechanisms underlying retinal dystrophies remain poorly understood. This has hampered the development of target-based drug screening strategies employed by the pharmaceutical industry.

The discovery of somatic cell reprogramming has enabled the generation of induced pluripotent stem cells (iPSCs) from adult tissue and allowed the use of patient-derived cells for in-vitro modelling of retinal disorders and cell replacement therapy (for reviews see [1–3]). Furthermore, CRISPR/Cas9 gene editing technology can be harnessed to repair the disease-causing mutation in patient-derived human iPSCs (hiPSCs) to generate unaffected and affected isogenic cell line pairs that will be critical for precise disease modelling [4, 5]. The resulting cells can then be differentiated into the retinal cell type of interest and the disease modelling carried out in a more reliable way than those solely utilising animal models, non-isogenic cell line pairs or overexpression/knockdown experiments. However, to use these advances for optimal in-vitro retinal disease modelling and cell transplantation studies requires protocols that result in efficient generation of retinal cells and maintenance of retinal architecture.

A landmark study by Eiraku et al. [6] paved the way for in-vitro generation of retinae from pluripotent stem cells. The study showed that mouse embryonic stem cells (mESCs) cultured in 3D suspension in the presence of extracellular matrix components spontaneously formed self-organising embryoid bodies that support the morphological differentiation of retinal cells. This so-called 3D culture technique has since been shown to sustain the differentiation of murine photoreceptors up to a stage equivalent to the second postnatal week [7, 8]. Several laboratories have also described the derivation of photoreceptors from human embryonic stem cells (hESCs) and hiPSCs using other approaches [9–19]. However, the generation of large numbers of bona-fide human photoreceptors for use in cell replacement therapy and disease modelling still presents several challenges. Importantly, existing methods of culture require extensive handling and are not suited to generate large quantities of cells. Stirred-tank bioreactors represent a simple, well-characterised platform that is widely used to produce large-scale bio-therapeutics, including, for example, human mesenchymal stem cells [20]. Cell transplantation to replace photoreceptors will require large batches of GMP cells and as a first step towards clinical application we tested whether we are able to adapt our recently described retinal differentiation protocol to small-scale (100 ml) spinner bioreactors.

Bioreactors provide cells with improved aeration and distribution of nutrients, as well as encouraging formation of complex structures. A number of studies have suggested that the use of bioreactors not only substantially increases the differentiation yield, but also improves the generation of a variety of 3D/suspension mini-organs or organoids derived from human pluripotent stem cells (hPSCs) when compared with conventional cell culture [21–30]. The modelling of retinal degeneration in vitro using iPS-derived cells from patients requires the generation of retinal organoids that faithfully generate specialised structures such as cilia and outer segments. Although a number of groups have demonstrated the development of these structures [19, 31–33], robust differentiation of mature outer segments with perfectly stacked membranous disks is yet to be achieved.

Here we coupled the use of bioreactors with our previously described retinal differentiation protocol [10] and found that the generation of retinal organoids under these conditions improved the preservation of retina-like architecture and enhanced the generation of photoreceptors. The presence of all major retinal cell types including neurons (photoreceptors, bipolar, horizontal, amacrine and RGCs) and Müller glia was observed. Moreover, we observed an increased number of photoreceptors bearing cilia and nascent outer-segment-like structures at an early stage of development. These results support the use of bioreactors for generating large quantities of retinal tissues for cell transplantation and disease modelling studies.

## Methods

### hPSC cell lines and culture conditions

The hPSCs used in this project were the WA09 H9 hESC line [34], the IMR90–4 hiPSC line and the research bank RB-002 H9 hESC line. These cells were obtained from WiCell Research Institute (Madison, VA, USA). hPSCs were grown and maintained in an undifferentiated state on Laminin-521 (Biolamina)-coated plates with E8 medium (Gibco). They were routinely passaged in six-well plates (Corning) at 1:6 ratios by incubating for 5–10 min with acutase at 37 °C. Acutase was washed off the cells by spinning them at 300 × *g* for 5 min and resuspending in fresh E8 medium. The resulting single cell suspension was subsequently plated into newly Laminin-521-coated six-well plates.

### hPSC retinal organoid differentiation

hPSCs grown on Laminin-521 were allowed to reach ~ 90% confluence in six-well plates (Corning) under self-renewing medium conditions. Once ~ 90% confluent, hPSCs were differentiated to retinal organoids as described by Gonzalez-Cordero et al. [10] with the following modifications. Briefly, after 4–5 weeks in culture, NRVs were transferred into either ultra-low-attachment 100-mm plates (Sarstedt) or 100-ml bioreactors (Chemglass) and cultured in RDM supplemented with 10% foetal bovine serum (Gibco), 2% Glutamax (Gibco) and 100 μM taurine (Sigma-Aldrich) (RDM + F) until formation of larger retinal organoids (weeks 5–10). BioMIXdrive 3 magnetic spinners (2Mag) were used to stir the medium in the bioreactors at a constant 22 rpm throughout the full differentiation period. The medium was changed once a week from here onwards. Developing retinal organoids were cultured in RDM + F supplemented with 1 μM retinoic acid (RA) (Sigma-Aldrich) (RDM + F + RA) (weeks 10–13). From week 13 onwards, retinal organoids where cultured with RDM + F made to the previous composition but using DMEM/F12 Glutamax (Cat. No. 10565–042; Gibco) instead of DMEM high glucose and adding 1% N2 supplement (RDM90 + F + RA), and the final retinoic acid concentration in culture was reduced to 0.5 μM (weeks 13–17).

### Immunohistochemistry

hPSC-derived retinal organoids were washed once in PBS and then fixed in 4% paraformaldehyde (PFA) for 30 min, and immersed in 20% sucrose/PBS at 4 °C overnight. The 20% sucrose/PBS was then removed completely, and the samples embedded in OCT matrix (Pyramid) and frozen in liquid nitrogen. Retinal organoid cryosections were cut 18 μm thick and all sections were collected for analysis. For immunohistochemistry, sections were blocked and permeabilised with PBS containing 0.3% Triton X-100, 10% goat serum and 1% bovine serum albumin (block/permeabilisation solution) for 1 h. Primary antibodies (Additional file 1: Table S1) were incubated overnight at 4 °C. Following primary antibody incubation, sections were incubated with Alexa-Fluor 488, 546 or 633 secondary antibodies (Invitrogen-Molecular Probes) in 1:500 dilutions at room temperature for 2 h, washed in PBS and counterstained with DAPI (Sigma-Aldrich). For Ki67 or Caspase 3-positive cell scoring, analysis of 10 random retinal neuroepithelia (ROIs of 275 μm^2^) per independent experiment was carried out (*n* = 10 ROIs, *N* = 3). ROI images are a series of *XY* optical sections, 1.0 μm apart, throughout the depth of the section built into a stack to give a projection image. Quantification of retinal layers involved masking the identity of each sample during analysis. Ten random regions of retinal neuroepithelia were assessed per independent experiment (*n* = 10 random regions of 10 retinal organoids, *N* = 3 independent experiments).

### Image acquisition

Images were acquired using a Leica DM5500Q confocal microscope. Leica LAS AF image software was used to take a series of *XY* optical sections, 1.0 μm apart, throughout the depth of the section and built into a stack to give a projection image.

### Flow cytometry

Fifty hPSC-derived retinal organoids per independent experiment were randomly selected and pooled together for tissue dissociation. Papain neurosphere dissociation kits (Miltenyi Biotec) were used, as per the manufacturer’s instructions. Samples were filtered through a 35-μm cell strainer to prevent cellular aggregation during acquisition. Single cell suspensions were spun down at 320 × *g* and stained with RECOVERIN, CD73 and CD133 antibodies. RECOVERIN staining was performed as described previously [10]. For CD133/CD73 co-staining, dissociated single cell samples were stained with anti-human CD73-PE and CD133-APC antibodies (Miltenyi Biotec) for 30 min on ice, in the dark, using DMEM^+^ media (10% FBS, 10 mM HEPES). The cells were then washed and resuspended in fresh DMEM^+^ media and stained with SYTOX Blue (Thermo Fisher Scientific) dead cell stain at a final concentration of 0.3 mM just before sample acquisition. For RECOVERIN/CD73 co-staining, dissociated single cell samples were stained with or without anti-human CD73-PE antibody (Miltenyi Biotec) and the LIVE/DEAD Fixable Violet Cell Stain (Thermo Fisher Scientific), for 30 min on ice, in the dark, using DMEM^+^ media. The cells were then washed with DMEM^+^ media and fixed in 2% PFA for 30 min on ice, in the dark. After the 30 min incubation, the cells were washed once with 1× PBS and then resuspended in BD permeabilisation/wash buffer (BD Biosciences) for 30 min on ice, in the dark. After permeabilisation, the cells were washed and resuspended in neat donkey serum (Biorad) for 10 min. Recoverin (Millipore) primary antibody was diluted 1:100 in permeabilisation buffer and added directly to the appropriate samples containing donkey serum. The cells were then washed with 1× PBS and resuspended in neat donkey serum for 10 min just prior to the addition of donkey anti-rabbit-AF647 secondary antibody (ThermoFisher). Samples were incubated for 30 min on ice, in the dark. After completion of the staining, the samples were washed and resuspended in 1× PBS. Just prior to sample acquisition, each sample was stained with DRAQ7 dead cell stain at a final concentration of 0.1 mM (Biostatus). Data were acquired on a five-laser BD LSRFortessa X-20 Analyser, equipped with 355 nm (UV), 405 nm (violet), 488 nm (blue), 561 nm (yellow) and 640 nm (red) lasers. Cells were analysed using FlowJo software. Background fluorescence compensation was accounted for using unstained cells and single-stained controls. Cell debris and dead cells were excluded from analysis on the basis of live–dead dye fluorescence followed by forward and side scatter.

### Serial block-face scanning electron microscopy (3view EM)

hPSC-derived retinal organoids were fixed in 3% glutaraldehyde and 1% paraformaldehyde in 0.08 M sodium cacodylate–HCl buffer, pH 7.4, and then stained en bloc with osmium ferricyanide–thiocarbohydrazide–osmium, uranyl acetate and Walton’s lead citrate with two modifications. First, the osmium concentration was reduced to 1% and, second, graded alcohols (50, 70, 90, 3 × 100%) and propylene oxide were used instead of acetone to dehydrate specimens for infiltration and curing overnight at 60 °C in Durcupan ACM resin. Specimens were then superglued to aluminium pins and trimmed to place the region of interest within a 0.5 mm × 0.5 mm × 0.4 mm mesa and sputter coated with 5 nm gold palladium. Stacks of backscatter electron micrographs were automatically acquired using a Gatan 3 view system working in conjunction with a Zeiss Sigma field emission scanning electron microscope working in variable pressure mode at a chamber pressure of 9 Pa and 4 kV. At a standard magnification of ×1000 and a pixel resolution of 4096 × 4096, the total area sampled measured 255.4 μm^2^ on *x* and *y*, and, depending on the number of 100-nm-thick sections sampled, between 67 and 150 μm on *z*. The resulting stacks were normalised for contrast and brightness and then converted to TIFF images in Digital Micrograph software prior to importation into Amira 5.3.3 software for semi-automated segmentation and presentation.

### Transmission and scanning electron microscopy

Sections (80 nm) for transmission electron microscopy were collected on formvar-coated grids and imaged without staining in a JEOL 11010 TEM operating at 80 V.

Images were acquired with a Gatan Orius camera using Digital Micrograph software.

For scanning electron microscopy, semi-thin sections 1 and 2 μm thick were cut and dried onto 20-mm glass coverslips, etched for 30 min in potassium methoxide (Cat. No. 60402, 250 ml; Sigma), rinsed twice for 5 min in methanol followed by hexamethyldisilazane (HMDS) and air dried. The dried specimens were coated with 1.5 nm of platinum and imaged in a Zeiss Sigma FESEM operating at 3–5 kV.

### Statistical analysis

Statistical tests were applied as specified in the text. All means are presented ± standard error of the mean (SEM), unless otherwise stated; *n* is the number of retinal organoids examined, *N* is the number of independent experiments performed. For quantification assessment by FC and cell counting, statistical analysis is based on at least three independent experiments. Statistical significance was assessed using GraphPad Prism 6 software and denoted as *P* < 0.05, *P* < 0.01 and *P* < 0.0001. Appropriate statistical tests were applied including an unpaired *t* test with Welch’s correction and two-way ANOVA.

## Results

### Bioreactor culture conditions increase photoreceptor cell yields

**Fig. 1:**
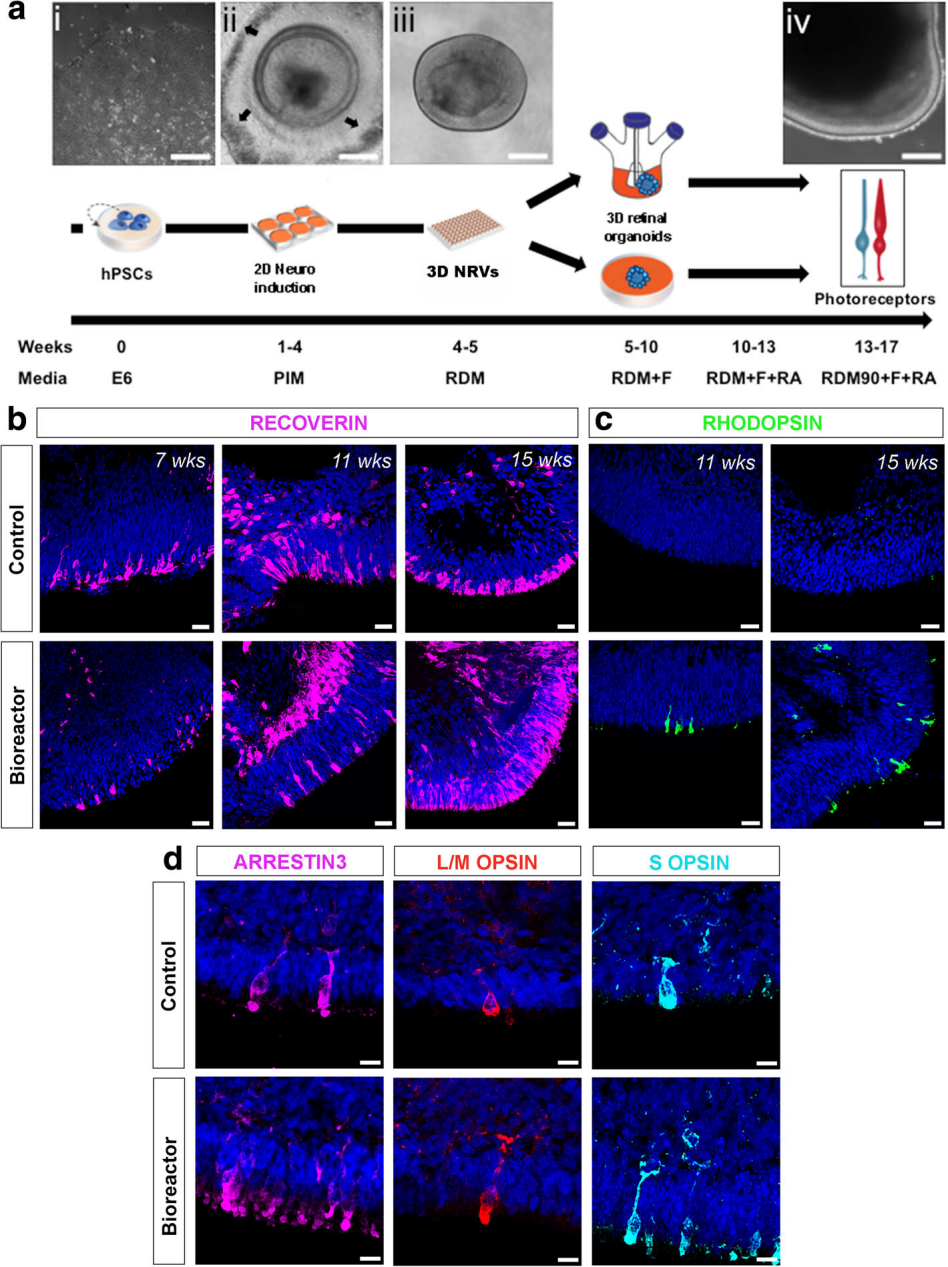
Photoreceptor maturation following bioreactor differentiation. **a** Schematic showing different stages of differentiation protocol. Representative phase-contrast images: (i) hPSCs at day 0 of differentiation; (ii) developing neuroepithelia surrounded by RPE cells (black arrows) at week 4; (iii) isolated retinal cup at week 5; (iv) mature retinal organoid at week 17. Following retinal cup formation, samples cultured in 100-mm cell culture plates or 100-ml bioreactors to form retinal organoids. Mature photoreceptors observed between days 90 and 120. **b, c** RECOVERIN and RHODOPSIN-positive photoreceptor cells in both control and bioreactor conditions at indicated number of weeks of differentiation. **d** Immunohistochemistry of L/M-OPSIN, Cone Arrestin (ARRESTIN3) and S-OPSIN cone markers at week 16 of differentiation in control and bioreactor conditions. Scale bars: 20 μm (a), 25 μM (b, c), 10 μM (d). hPSC human pluripotent stem cell, NRV neuroretinal vesicle, RDM + F RDM supplemented with 10% foetal bovine serum + 2% Glutamax + 100 μM taurine, RA retinoic acid

Recently, we have adapted some of the human retinal differentiation protocols described previously [18, 19] and demonstrated the generation of hPSC-derived neuroretinal vesicles (NRVs) containing photoreceptors with nascent outer segment (OS)-like structures [10]. Here, we used this protocol to differentiate hPSC lines (WA09 H9 and IMR90-4) into retinal organoids, using bioreactor technology. A schematic of this in-vitro retinal differentiation approach is shown in Fig. 1a. Briefly, hPSCs were cultured on Laminin-521-coated plates (Fig. 1ai). Neuroretinal domains appeared towards the fourth week of differentiation; they were easily identifiable by surrounding patches of retinal pigment epithelium (RPE) cells (Fig. 1aii, arrows). Neuroretinal structures were dissected together with patches of RPE cells and grown as NRVs in 3D suspension cultures (Fig. 1aiii, aiv). We previously described that photoreceptor markers start to be expressed as early as 6 weeks of culture [10]. Therefore, at 5–6 weeks of culture a total of 50 NRVs were randomly allocated per independent experiment to either 100-mm ultra-low-attachment plates (control) or 100-ml bioreactors (see Methods for further details). According to our previous data [10] we expect that out of the randomly allocated NRVs only about 10% will be non-retinal.

We sought to determine whether bioreactors improve retinal differentiation. For clarity, following their placement in bioreactors, we refer to NRVs as retinal organoids. The size of retinal organoids varied in both conditions, with bioreactor organoids being larger on average (1783 mm^2^ ± 1453) than control samples (1115 mm^2^ ± 644) (*n* = 15 organoids, *N* = 2 independent experiments). We observed that the morphology of retinal organoids in both control and bioreactor conditions can change during differentiation, with some organoids forming rosettes and becoming disorganised. Organoids grown in bioreactors sometimes fuse to each other, forming a large single organoid.

Next, we compared photoreceptor differentiation at weeks 7, 11, 15 and 16 of culture in controls vs bioreactor conditions. RECOVERIN-positive photoreceptor cells were present in both cultures at all time points analysed (Fig. 1b). Maturing, RHODOPSIN-positive, rod photoreceptors first appeared at week 11 under bioreactor conditions and at week 15 under control conditions (Fig. 1c). Furthermore, both control and bioreactor samples contained S-OPSIN, L/M-OPSIN and cone ARRESTIN-positive cone photoreceptors (Fig. 1d). By week 16 of differentiation both protocols resulted in expression of mature markers for both rod and cone photoreceptors.

**Fig. 2:**
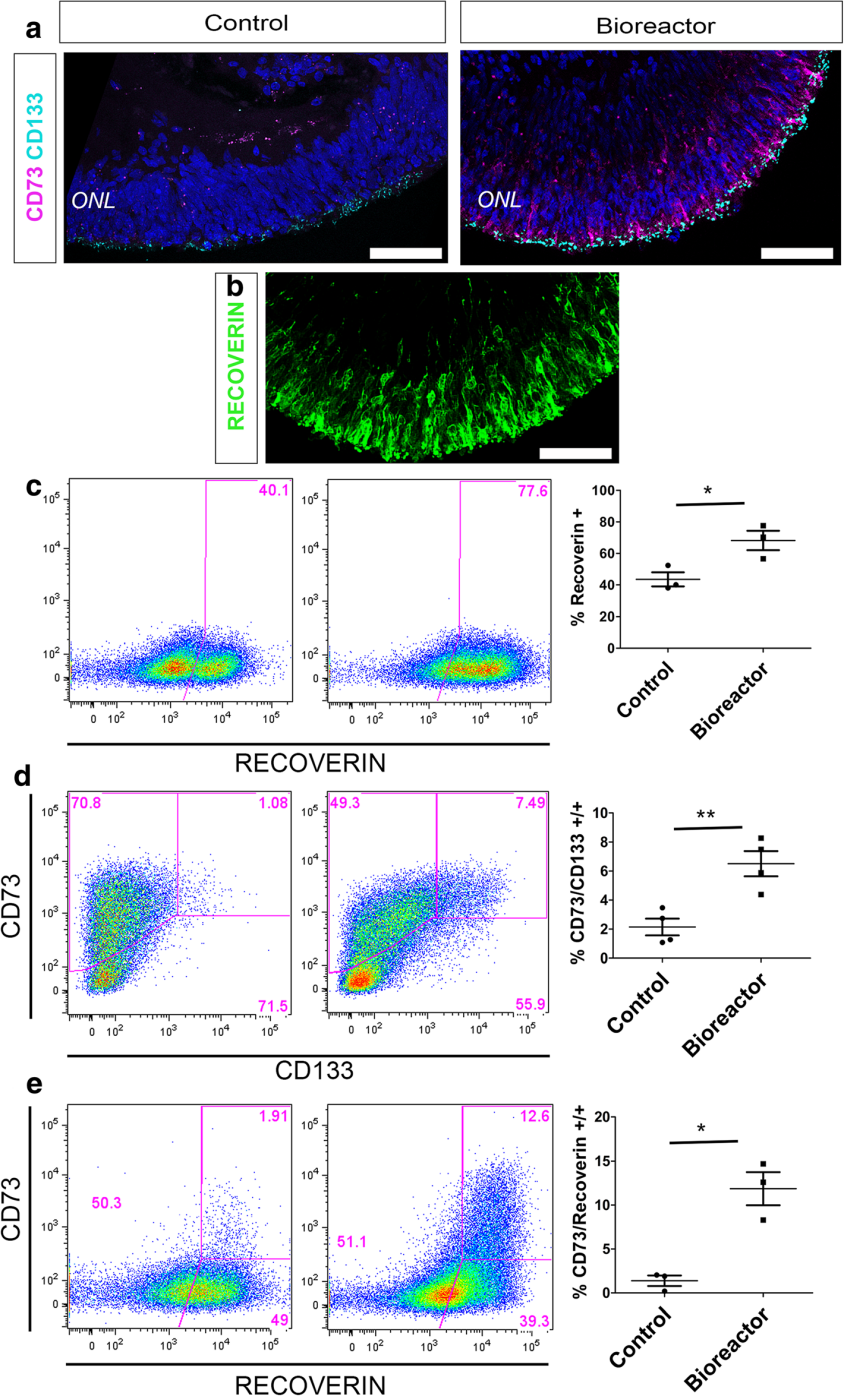
Photoreceptor quantification following bioreactor differentiation. **a, b** Cryosections from week 16 bioreactor cultures showing both CD73 and CD133 cell surface markers localised to ONL-like region **(a)** where RECOVERIN-positive photoreceptors are also located **(b)**. **c–e** Representative FC analysis of week 16 bioreactor cultures vs control cultures: RECOVERIN-positive photoreceptor cells (68.17% ± 6.15 vs 43.53% ± 4.47) **(c)**; CD133/CD73 double-positive developing rods (6.50% ± 0.86 vs 2.14% ± 0.57) **(d)**; RECOVERIN/CD73 mature photoreceptors (11.87% ± 1.88 vs 1.38% ± 0.59) **(e)**. Error bars, mean ± SEM; *n* = 50 retinal organoids per individual FC experiment, *N* = 3–4 independent differentiation experiments; *P < 0.05, **P < 0.01, two-tailed unpaired *t* test with Welch’s correction. Scale bars: 50 μM (a, b). ONL outer nuclear layer

Next, to quantify the number of photoreceptors present in both control and bioreactor conditions we used flow cytometric (FC) analysis on week 16 retinal organoids. We and others have demonstrated that cluster of differentiation CD73 and CD133 cell surface markers are expressed in the photoreceptors of foetal and adult human retina and can be used to delineate developing rods (CD133/CD73 co-labelled cells) and mature photoreceptors (CD73) [35–37]. Therefore, we used these markers together with RECOVERIN staining to quantify photoreceptor yield in our cultures. We confirmed the specificity of these markers to the hPSC-derived photoreceptor cells by immunohistochemistry (IHC) in control and bioreactor organoids at 16 weeks of development. Both CD73 and CD133 staining was localised to the ONL-like structure of the organoids (Fig. 2a), where RECOVERIN-positive photoreceptors are also located (Fig. 2b, bioreactor representative image). However, in agreement with FC results, CD73 staining was much more evident in bioreactor-cultured organoids.

FC analysis showed a significant increase in the proportion of RECOVERIN-positive cells in retinal organoids under bioreactor conditions (68.17% ± 6.15) vs control cultures (43.53% ± 4.47) (Fig. 2c, *n* = 50 retinal organoids analysed together, *N* = 3 independent experiments; *P* < 0.05). RECOVERIN is also expressed in other retinal cells such as cone bipolar cells. Therefore, to precisely quantify photoreceptors we co-stained organoids with CD133/CD73 and RECOVERIN/CD73. Again, we observed significant increase in the number of CD133/CD73 double-positive photoreceptors under bioreactor conditions (6.50% ± 0.86) in comparison to control retinal organoids (2.14% ± 0.57) (Fig. 2d, *n* = 50 retinal organoids, *N* = 4 independent experiments; *P* < 0.01). To quantify the proportion of developing photoreceptors within the photoreceptor population only, we gated on the CD133 photoreceptor population and quantified the number of CD73-positive cells within this population. There was a significant increase from 3.23% ± 0.95 in controls to 9.98% ± 1.37 in bioreactors (Additional file 1: Figure S1A, *n* = 50 retinal organoids, *N* = 4 independent experiments; *P* < 0.01). RECOVERIN/CD73 co-staining and FC analysis revealed a discrete 1.38% ± 0.59 double-positive population in the control organoids while a more abundant 11.87% ± 1.88 population was observed in the bioreactor samples (Fig. 2e, *n* = 50 retinal organoids, *N* = 3 independent experiments; *P* < 0.05). Next, to analyse the proportion of mature photoreceptors within the photoreceptor population only, we gated RECOVERIN-positive cells and calculated the percentage of CD73-positive cells within this population. A significant increase in mature CD73-positive photoreceptors was observed in bioreactor samples (15.83% ± 2.52) when compared to controls (5.78% ± 1.64) (Additional file 1: Figure S1B, *n* = 50 retinal organoids, *N* = 3 independent experiments; *P* < 0.05). All gating strategies and pertinent controls are shown in Additional file 1: Figure S2. Low magnification imaging of whole organoid RECOVERIN-stained sections supported the increased presence of photoreceptors (Additional file 1: Figure S3). It is important to note that at this early time point in culture (16 weeks) the majority of photoreceptors cells are still precursors and therefore only a small percentage of the RECOVERIN-positive cells express the mature CD73 marker.

### Improved retinal organoid lamination is observed under bioreactor conditions

**Fig. 3:**
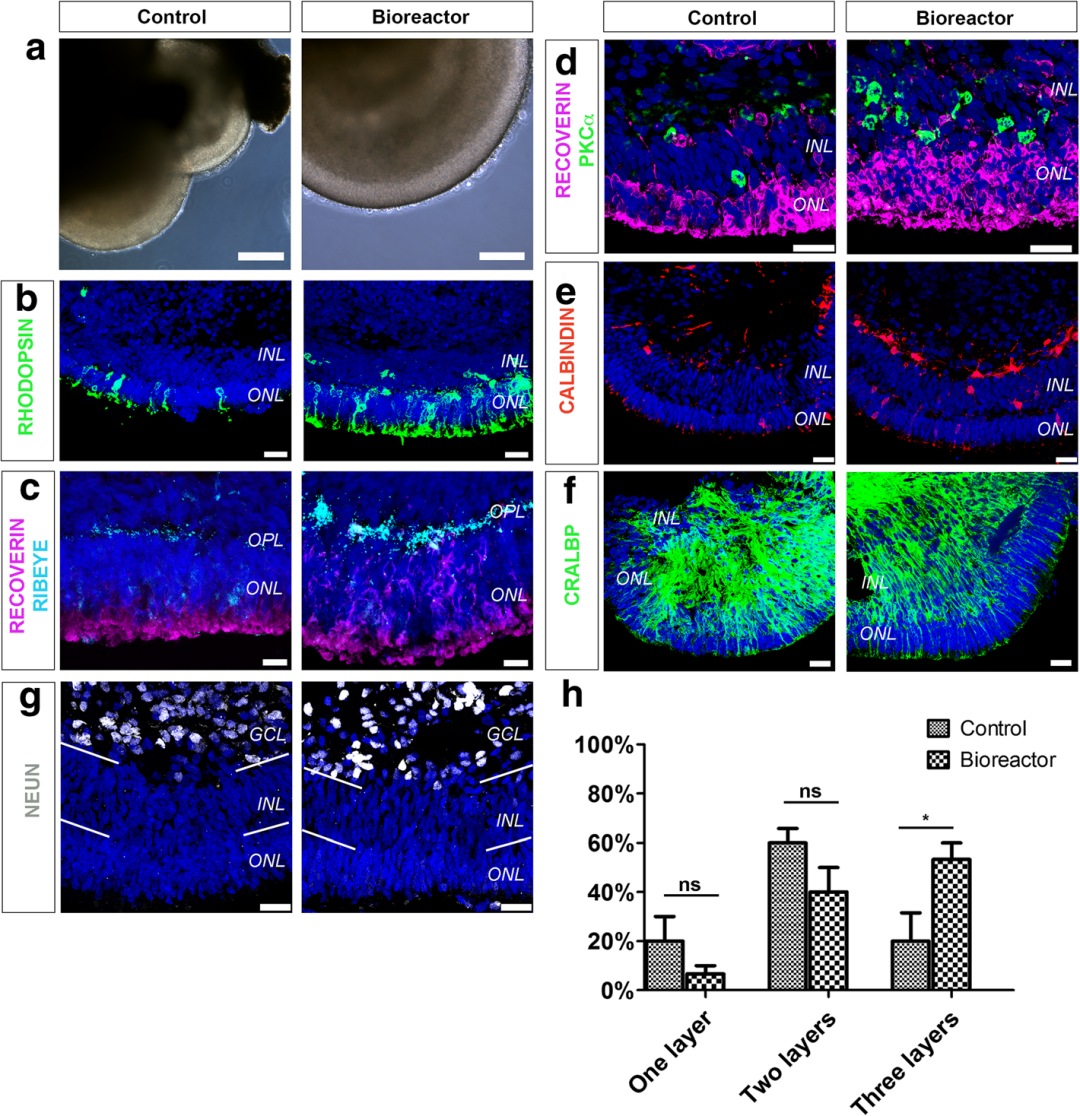
Characterisation of neuroepithelia lamination. **a–f** Phase-contrast images **(a)** and immunohistochemical analysis **(b–f)** of week 16 hPSC-derived retinal organoids grown in bioreactors and in control conditions. RHODOPSIN-positive **(b)** and RECOVERIN-positive photoreceptors in ONL and RIBEYE-positive ribbon synapses in OPL **(c)**. PKCα-positive rod bipolar cells **(d)** and CALBINDIN-positive amacrine and horizontal interneurons in INL **(e)**. CRALBP-positive Müller glia cells also present in both culture conditions **(f)**. **g** Presence of NEUN-positive RGCs and subset of amacrine cells. **h** Quantification of number of retinal layers in control and bioreactor retinal organoids following DAPI staining. *n* = 10 random regions of retinal neuroepithelia per independent experiment, *N* = 3 independent experiments. Error bars, mean ± SEM; ns, *P* > 0.05; *P < 0.05, two-way ANOVA. Scale bars: 25 μM (a, b, d–g), 10 μM (c). GCL ganglion cell layer, INL inner nuclear layer, ns not significant, ONL outer nuclear layer, OPL outer plexiform layer

Brain organoid studies have demonstrated that bioreactors grow more complex structures than static methods of culture [23, 25, 26, 29]. Phase-contrast images of week 16 control and bioreactor retinal organoids showed retinal epithelium layering in both samples. To carefully establish the morphology of retinal epithelium we examined retinal lamination by IHC (Fig. 3). Three clearly defined layers form the neural retina: the outer nuclear layer (ONL) containing photoreceptors, the inner nuclear layer (INL) containing interneurons and Müller glia cells, and a ganglion cell layer (GCL). Structures reminiscent of these layers were present in both culture conditions. RHODOPSIN and RECOVERIN-positive cells delineated the ONL-like structure in control and bioreactor conditions (Fig. 3b, c). Pre-synaptic marker RIBEYE was detected under bioreactor and control conditions between the ONL and INL at the outer plexiform layer (OPL), (Fig. 3c). To distinguish the INL, we performed IHC for markers of interneurons. PKCα-positive bipolar cells were found at the INL-like layer in both samples (Fig. 3d). In addition, CALBINDIN-positive horizontal and subset of amacrine cells were also present in both cultures (Fig. 3e). The presence of CRALBP-positive Müller glia cells was also evident (Fig. 3f).

Next, we analysed the differentiation of the GCL. Both control and bioreactor cultures revealed a GCL as shown by NEUN staining (Fig. 3g). Finally, we carried out quantification of the number of layers in the retinal neuroepithelium, where one layer indicates the presence of an ONL, two layers indicate the presence of an ONL and an INL, and three layers indicate the presence of an ONL, an INL and GCL-like layers. We observed a significant difference in the number of retinal neuroepithelia with three retinal layers. In retinal neuroepithelia cultured in bioreactors 53.33% ± 6.66 comprised three layers, compared with 20% ± 11.55 in control neuroepithelia (Fig. 3h, *n* = 10 neuroepithelium regions, *N* = 3 independent experiments; ns, *P* > 0.05 and *P < 0.05). This increase was offset by a non-significant decrease in the number of control and bioreactor retinal neuroepithelia bearing one or two retinal layers (Fig. 3h).

### A decrease in apoptotic cells and increased cell proliferation are observed in bioreactor retinal organoids

**Fig. 4:**
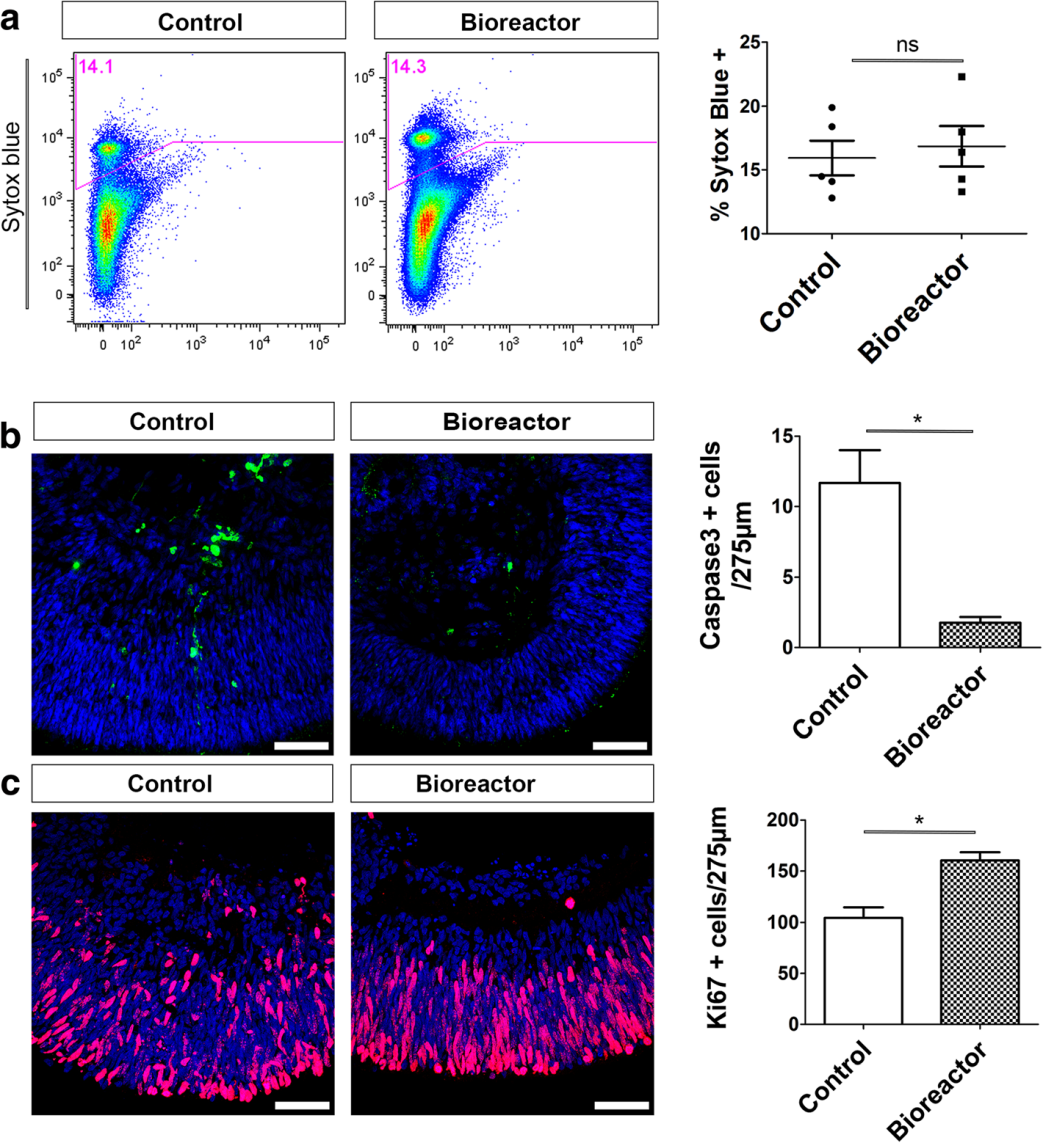
Bioreactor culture of retinal organoids increases cell proliferation and reduces cell death. **a** FC analysis of week 16 control vs bioreactor hPSC-derived retinal organoids stained with SYTOX Blue (error bars, mean ± SEM; *n* = 50 retinal organoids, *N* = 5 independent experiments; ns, *P* > 0.05, two-tail unpaired *t* test with Welch’s correction). **b** Immunohistochemistry analysis and quantification of cleaved caspase-3 retinal organoids showing reduction in number of caspase-3-positive apoptotic cells (error bars, mean ± SEM; *n* = 10, *N* = 3; *P < 0.05, two-tail unpaired *t* test with Welch’s correction). **c** Immunohistochemistry and quantification of Ki67 proliferative cells revealed significant increase in number of proliferating cells present in neuroepithelia of bioreactor cultures (error bars, mean ± SEM; *n* = 10 images, *N* = 3 independent experiments; *P < 0.05, two-tail unpaired *t* test with Welch’s correction). ns not significant

Next, we aimed to establish the reasons behind the increase in photoreceptor cell numbers observed in bioreactor cultures. Spinning bioreactors are thought to improve culture conditions by promoting oxygenation and to facilitate nutrient absorption, features that in turn can regulate cell viability and proliferation. FC analysis of retinal organoids was used to assess cell viability. The proportion of dead cells was established by SYTOX Blue uptake. No significant differences in whole retinal organoid cell death content were observed. Control retinal organoids contained 15.94% ± 1.36 dead cells while the bioreactor samples contained 16.86% ± 1.58 dead cells (Fig. 4a, *n* = 50 organoids analysed together, *N* = 5 independent experiments; ns, *P* > 0.05). We then used cleaved Caspase-3 IHC to specifically assess the presence of apoptotic cells in regions of neuroepithelium from week 16 retinal organoids. Quantification of Caspase-3-positive cells showed an average of 1.76 ± 0.41 cells/275 μm^2^ under bioreactor conditions which significantly increased to 12.00 ± 2.00 cells/275 μm^2^ in control conditions (Fig. 4b, *n* = 10 neuroepithelium regions, *N* = 3 independent experiments; *P* < 0.05). Previous reports using bioreactors to differentiate hPSCs have shown increased yields of mature cell types under these conditions [21, 22, 27, 28, 30]. We previously described that the retinal epithelia of retinal organoids were proliferative at an early stage in cultures, with proliferation ceasing by 12 weeks of development [10]. Here, we compared control vs bioreactor retinal organoids at 8 weeks for the presence of Ki67-positive cells. At this time point retinal organoids had been in the bioreactor vessels for 3 weeks. Retinal epithelium of control organoids contained 104 ± 10.4 cells/275 μm^2^ while the number of proliferative cells increased significantly in bioreactor organoids to 160 ± 7.932 cells/275 μm^2^ (Fig. 4c, *n* = 10 neuroepithelium regions, *N* = 3 independent experiments; *P* < 0.05). These results suggest that the increased number of photoreceptors observed in bioreactors is due to a synergistic combination of increased proliferation during the early differentiation stage and a reduction in cell death at the later time points.

### Bioreactor-differentiated retinal organoids have a greater number of photoreceptors with nascent outer segments

**Fig. 5:**
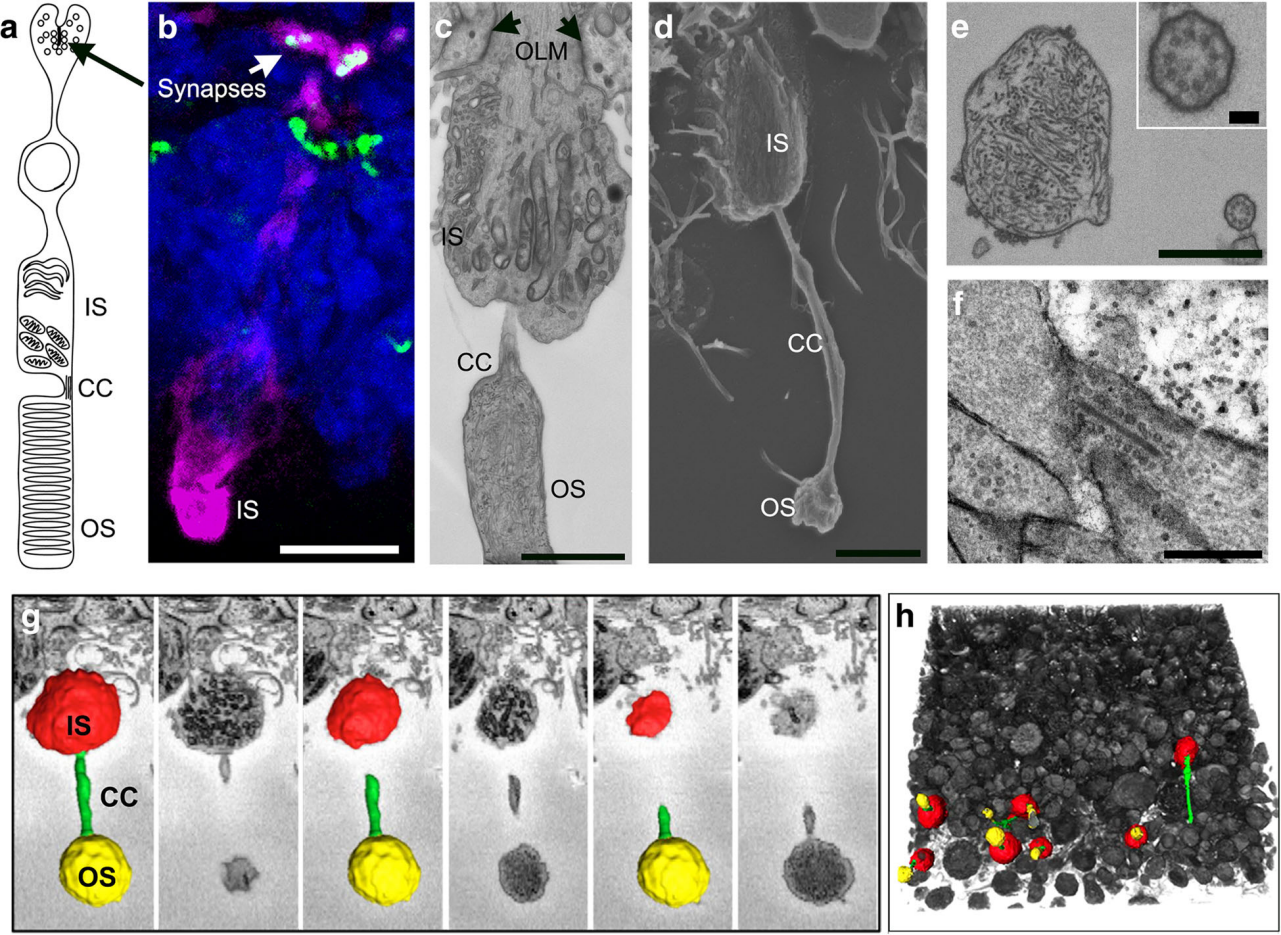
Ultrastructural and topographical analysis of human hPSC-derived neuroepithelia and photoreceptor cells at early stages of development. **a** Diagrammatic representation of key structures of mature photoreceptor. **b** Immunohistochemical staining of cone photoreceptor (L/M-OPSIN, magenta) and its ribbon synapse (RIBEYE, green, arrow). **c** TEM image illustrating morphology of hPSC-derived photoreceptor OLM (arrows), IS, CC and OS-like structures. **d** SEM image from etched resin section 2 μm thick showing IS, CC and OS. **e** TEM images illustrating internal organisation of CC (high magnification box) and OS. **f** Backscatter EM images from different levels of 3view dataset showing photoreceptor ribbon synapse. **g** 3view sequence of backscatter EM images of hPSC-derived photoreceptor OS (yellow), CC (green) and IS (red). See Additional file 2: Movie 1. **h** 3view 3D reconstruction of 150 sections with thickness of 100 nm each. Representative photoreceptor OS, CC and IS observed in yellow, green and red respectively. CC connecting cilium, IS inner segment, OLM outer limiting membrane, OS outer segment


Mature photoreceptors can be recognised by the presence of an OS and a ribbon synapse. The presence of these features is key for assessing the functionality of photoreceptors in vitro for disease modelling and drug screening purposes. A schematic of a photoreceptor cell and the PSC-derived in-vitro counterpart are shown in Fig. 5a, b, respectively. Here we used transmission electron microscopy (TEM), scanning electron microscopy (SEM) and 3view electron microscopy to investigate the ultrastructural content and topography of photoreceptors, as well as their organisation in 3D. TEM and SEM analysis of week 16 bioreactor-cultured retinal organoids revealed several features reminiscent of the human outer retina, including an outer limiting membrane and an organised layer of photoreceptors comprised of inner segments containing mitochondria, connecting cilium (CC) and developing outer segments (Fig. 5c, d and Additional file 1: Figure S4). TEM showed that the CC of these photoreceptors contained organised 9 + 0 microtubule pairs, lacking a central microtubule, typical of primary (non-motile) sensory cilia, and an OS structure containing intracellular membranes reminiscent of photoreceptor OS discs (Fig. 5e). Photoreceptor ribbon synapses were also observed (Fig. 5f). To analyse in detail the retinal organoid photoreceptor layer, we used 3view EM, which allows for 3D reconstruction of serial EM sections. We reconstructed 150 sections and pseudo-coloured specific regions of representative photoreceptors. Inner segments were identified by their characteristically high tightly packed mitochondrial content and labelled red, connecting cilia were detected by their elongated axonemes and microtubule content and labelled green, and the outer segments, labelled yellow, were identified by their content of intracellular membranes (Fig. 5g). A low-magnification image of this reconstruction showed many preserved inner segment (IS)/CC/OS structures at the apical edge of bioreactor retinal organoids (Fig. 5h and Additional file 2: Movie 1). At week 16 of development, there was a significant increase in the number of maturing photoreceptor structures (CC–OS-like structures) in organoids cultured in bioreactors (149.7 ± 8.69/125 μm^2^) compared to control samples (8.33 ± 2.85/125 μm^2^) (*n* = 4 SEM images, *N* = 3 independent experiments; *P* < 0.01).

**Additional file 2: Movie 1.** 3view EM reconstruction. Video showing 3view serial block-face scanning electron microscopy reconstruction of photoreceptor region illustrating photoreceptor inner segments (red), connecting cilia (green) and developing outer segments (yellow) of a bioreactor retinal organoid (MP4 22,030 kb)


**Fig. 6:**
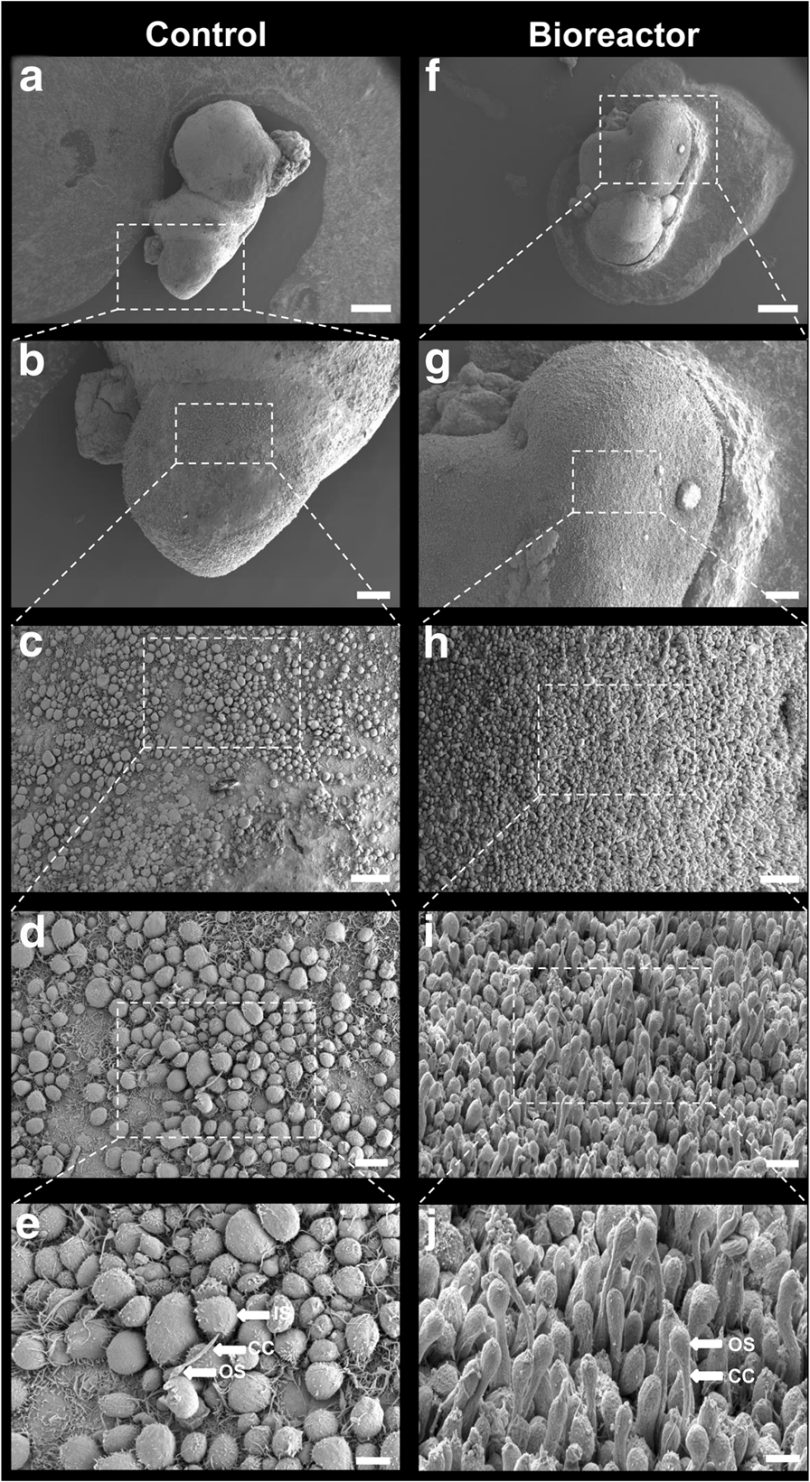
SEM images showing topography of whole retinal organoids. Topographic features of neuroepithelia showing photoreceptor cell density and morphology from control **(a–e)** vs bioreactor **(f–j)** at ascending magnifications. Scale bars: 400 μM (a, f), 100 μM (b, g), 30 μM (c, h), 10 μM (d, i), 5 μM (e, j). CC connecting cilium, IS inner segment, OS outer segment

Representative EM images show bioreactor organoids have denser regions of photoreceptors containing CC and nascent OS-like structures (Additional file 1: Figure S4D, Fig. 6 and Additional file 1: Figure S5, *n* = 5 retinal organoids, *N* = 3 independent experiments).

**Fig. 7:**
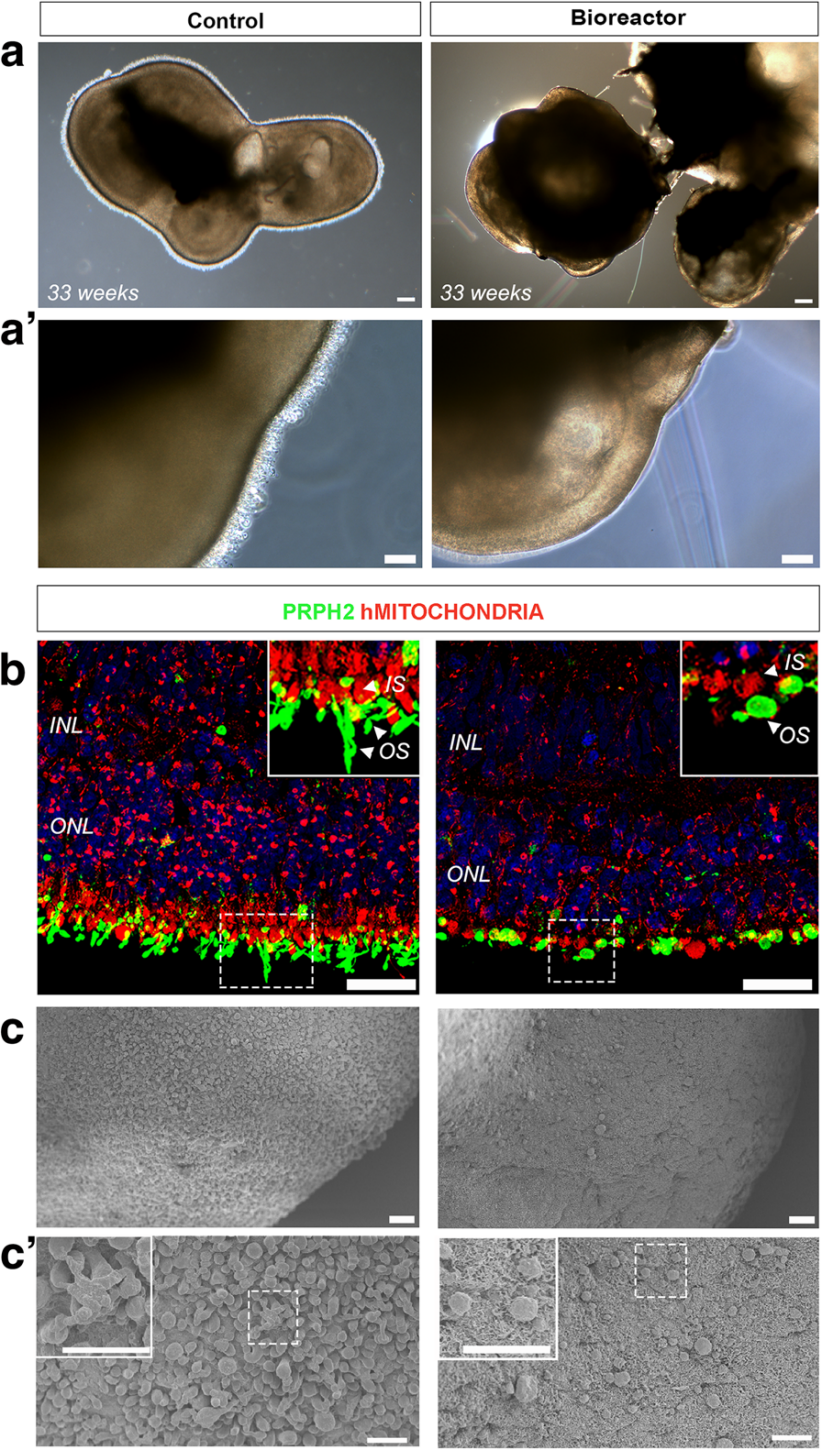
Characterisation of human hPSC-derived neuroepithelia and photoreceptor cells at late stages of development. **a** Representative bright-field images of retinal organoids cultured in standard plates and bioreactors. **a′** High-magnification images of neuroepithelia region showing CC/OS brush border in control but not in bioreactor samples. **b** Immunohistochemistry analysis for hMITOCHONDRIA and PRPH2 to delineate IS and OS, respectively, showed elongated OS-like structures in control samples only. **c, c′** SEM images showing the topography of whole retinal organoids. Photoreceptor cell density and morphology from control vs bioreactor at ascending magnifications. Scale bars: 200 μM (a), 100 μM (a′), 25 μM (b), 20 μM (c), 10 μM (c′ and high magnification box). INL inner nuclear layer, IS inner segment, ONL outer nuclear layer, OS outer segment

Next, we sought to establish whether OS development under bioreactor conditions was improved at later developmental time points. We and others have previously demonstrated the formation of a brush-like border surrounding the retinal epithelium [10, 31]. These regions contain the cilia and OS-like structures observed under electron microscopy. Bright-field images of week 33 retinal organoids showed that only control samples displayed well-developed brush borders (Fig. 7a, a′, *n* = 50 retinal organoids, *N* = 4 independent experiments). To confirm whether bioreactor samples were depleted of cilia and nascent outer segments, we performed IHC using a human MITOCHONDRIA antibody that highlights the mitochondria-rich region of the inner segments and a peripherin (PRPH2) (Fig. 6b) antibody that is a marker of outer segments. Similar to the bright-field images, elongated OS-like structures apical to the inner segments were only observed in the control samples. Similarly better preservation of IS and OS-like structures in the control samples was also observed in SEM samples (Fig. 7c, c′). These results suggest that the spinning bioreactor environment improves the integrity of inner segments, connecting cilia and nascent OS-like structures at early but not later stages of development.

## Discussion

The use of bioreactors for the large-scale culture of stem cells, including mesenchymal and pluripotent stem cells, has been studied extensively [38, 39]. Recently, suspension cultures using bioreactors have been used to generate and maintain mini-organs, including complex brain structures [26]. These organoids are now being used to further understand the pathophysiology of many human diseases. In this study, we tested for the first time the differentiation of hPSC-derived retinal organoids using a bioreactor platform. We found that this culture approach improved retinal organoid stratification as well as the yield of photoreceptors, possibly due to increased proliferation and a decrease in cell apoptosis. Furthermore, at 16 weeks there was an increase in the number of photoreceptors bearing cilia and OS-like structures following culture in bioreactors.

The differentiation of hPSCs into retinal organoids that closely resemble the human retina has been demonstrated previously. Furthermore, GMP-compliant protocols for the differentiation of retinal cells have been developed [40, 41]. Also of importance for clinical applications is the development of protocols that can generate retinal cell types in sufficient numbers to permit the routine isolation of these cells for transplantation. We previously demonstrated the generation of photoreceptor cells in static 3D suspension cultures [10]. Our protocol generated a sufficient number of cone photoreceptor cells that could be purified and transplanted into mouse models of retinal degeneration. An average of 1 million cells could be isolated from around 120 retinal organoids cultured in 24-well plates. These relatively small-scale cultures were sufficient for our purposes as only a few thousand cells were required for transplantation in the mouse eye. However, to meet future requirements for transplantation into the human eye, a system that allows the culture of a large number retinal organoids is required. Further studies are required to optimise the adherent culture phase and, importantly, the manual, highly laborious and time-consuming separation of NRVs. The ideal platform would sustain a large number of healthy cells as well as minimise personnel and culture costs. In this study, we established that bioreactor technology is able to sustain the differentiation of retinal organoids within a normal human developmental window [42–47] and, most importantly, increase the yield of CD73/RECOVERIN double-positive photoreceptor cells when compared to static culture conditions.

Prolonged culture periods are a feature of retinal organoid differentiation protocols as development can take many months. This can sometimes lead to morphological disorganisation and cell death. We have demonstrated that our bioreactor conditions support the survival of retinal cells and promote a significant improvement in the maintenance of lamination within retinal organoids. We suspect that the observed improvement in maintenance and differentiation of retinal organoids is a result of a significant increase in cell proliferation and reduced cell death in the retinal epithelium of the bioreactor-derived retinal organoids. This is consistent with previous reports using bioreactors for the differentiation of brain organoids [26] and other tissues [21, 22, 27, 28, 30] that have demonstrated an improvement in cell expansion, structure and differentiation.

Retinal organoids may also provide useful tools for disease modelling studies. Despite substantial progress in developing retinal differentiation protocols, to date the formation of mature photoreceptor outer segments containing stacked membrane disks has proved elusive. Here we aimed to evaluate the development of CC and OS structures when organoids are cultured in bioreactors. Photoreceptor cells grown in bioreactors developed nascent CC and OS structures at later stages, and the retinal organoids lost the brush border regions comprising the OS-like structures. These structures are very fragile and their loss was possibly due to continuous exposure to shear stress in the stirred bioreactor system. Further optimisation of the bioreactor process is needed regarding the optimal stirring rate in order to maintain a balance between maximum cell yield and the preservation of fragile structures. It will also be important to test different feeding regimes with the aim of minimising handling and media costs and at the same time avoiding the depletion of nutrients and the accumulation of metabolic by-products.

## Conclusions

In summary, we have shown that bioreactor culture of retinal organoids improves laminar stratification as well as the yield of photoreceptor cells bearing cilia and nascent OS-like structures. Our results support the further development of bioreactors for scaling up the manufacture of retinal cells.

## Additional files

Additional file 1: **Figure S1.** Flow cytometric analysis and quantification of proportion of RECOVERIN/CD73 and CD133/CD73 double-positive cells within RECOVERIN and CD133 photoreceptor-positive populations. Representative FC plots of control vs bioreactor retinal organoids. **A** FC quantification of CD133/CD73 double-positive developing rods within CD133-positive population. **B** Quantification of RECOVERIN/CD73 double-positive mature photoreceptor cells by gating only in RECOVERIN-positive live cell population. Error bars, mean ± SEM; *n* = 50 retinal organoids, *N* = 3–4 independent differentiation experiments carried out per control or bioreactor condition; *P < 0.05, **P < 0.01, two-tail unpaired *t* test with Welch’s correction. **Figure S2.** Flow cytometry gating strategy employed for all flow cytometric analysis for each individual sample. **A** Dead cells excluded by using DRAQ7 vs FSC-A (or SYTOX Blue vs FSC-A; data not shown). Cellular aggregates gated out (FSC-A vs FSC-H) to ensure only single live cells (SSC-A vs FSC-A) used for subsequent analysis. **B** Representative plots of control vs bioreactor for RECOVERIN staining. Gates drawn using only secondary control samples for both control and bioreactor samples. **C** Representative plots of gating strategy used for CD73 staining in combination with CD133 antibody staining for both control and bioreactor samples. Unstained and fluorescence minus one (FMO) controls for CD73 and CD133 used to define positive fraction of cells for both control and bioreactor samples. **D** Representative plots for RECOVERIN and CD73 staining. Unstained and FMO gating controls used to determine RECOVERIN and CD73-positive cells for both control and bioreactor samples. **Figure S3.** Immunofluorescence analysis showing Müller glia (CRALBP-positive) and photoreceptor (RECOVERIN-positive) cells of week 15 retinal organoids in control (**A**) and bioreactor (**B**) conditions. Scale Bars: 200 μM. **Figure S4.** SEM and TEM images of hPSC-derived retinal organoid OLM regions. **A, B** SEM image showing photoreceptors of bioreactor-generated retinal organoid. **C**, **D** TEM illustrating photoreceptor outer limiting membrane (OLM), inner segments, CC and developing outer segments of control (**C**) and bioreactor (**D**) retinal organoids. Scale bars: 2 μm (B–D). **Figure S5.** SEM images of whole retinal organoid. Topographic features of neuroepithelia showing photoreceptor cell density and morphology from control (**A–C**) vs bioreactor (**E–G**) at ascending magnifications. Scale bars: 10 μM. **Table S1.** Antibody catalogue numbers and dilutions (DOCX 8526 kb)
