# Supplementary figures and images for: Use of bioreactors for culturing human retinal organoids improves photoreceptor yields
Source: Stem Cell Res Ther. 2018 Jun 13;9:156. doi: 10.1186/s13287-018-0907-0 (PMC5998504; doi:10.1186/s13287-018-0907-0)

**Figure S1.**


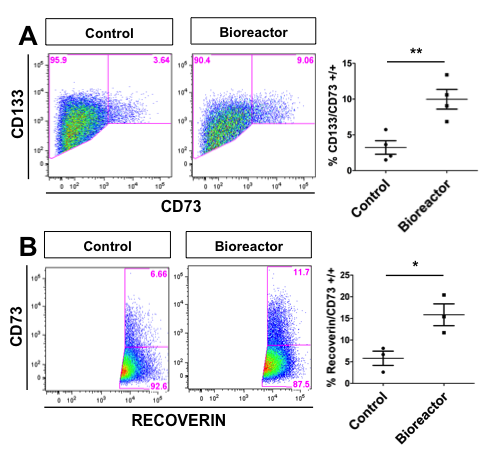


**Figure S2.**


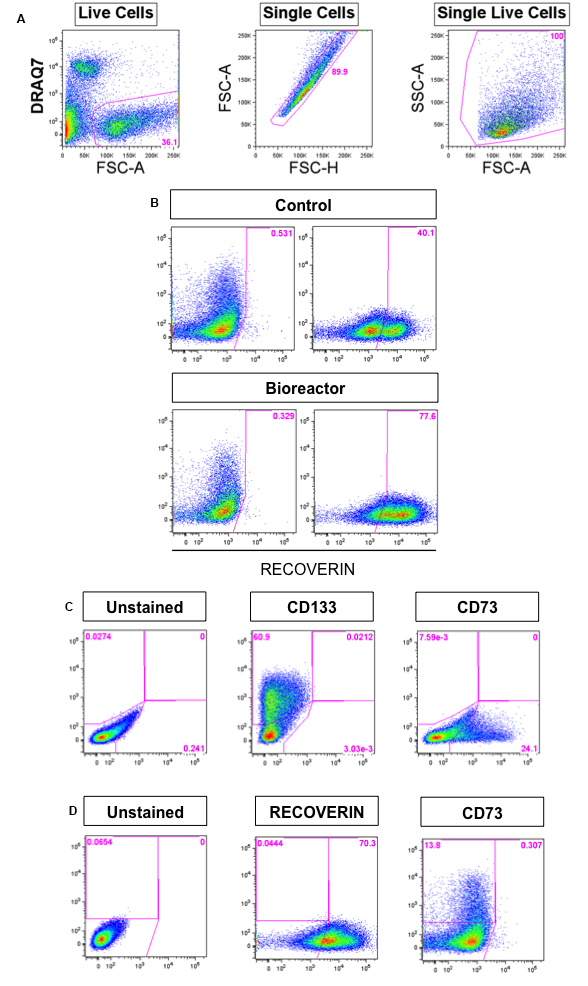


**Figure S3.**

**
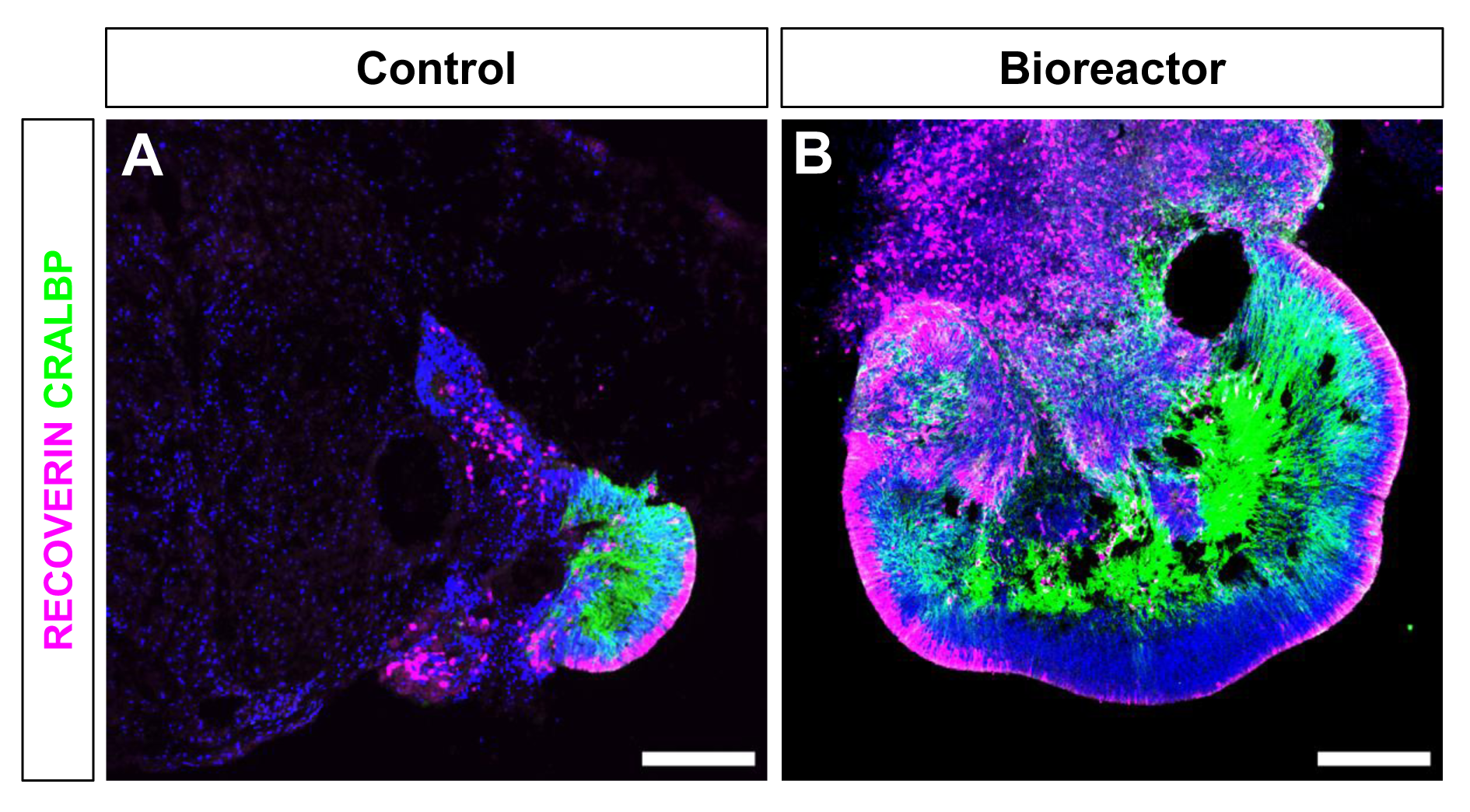
**

**Figure S4.**


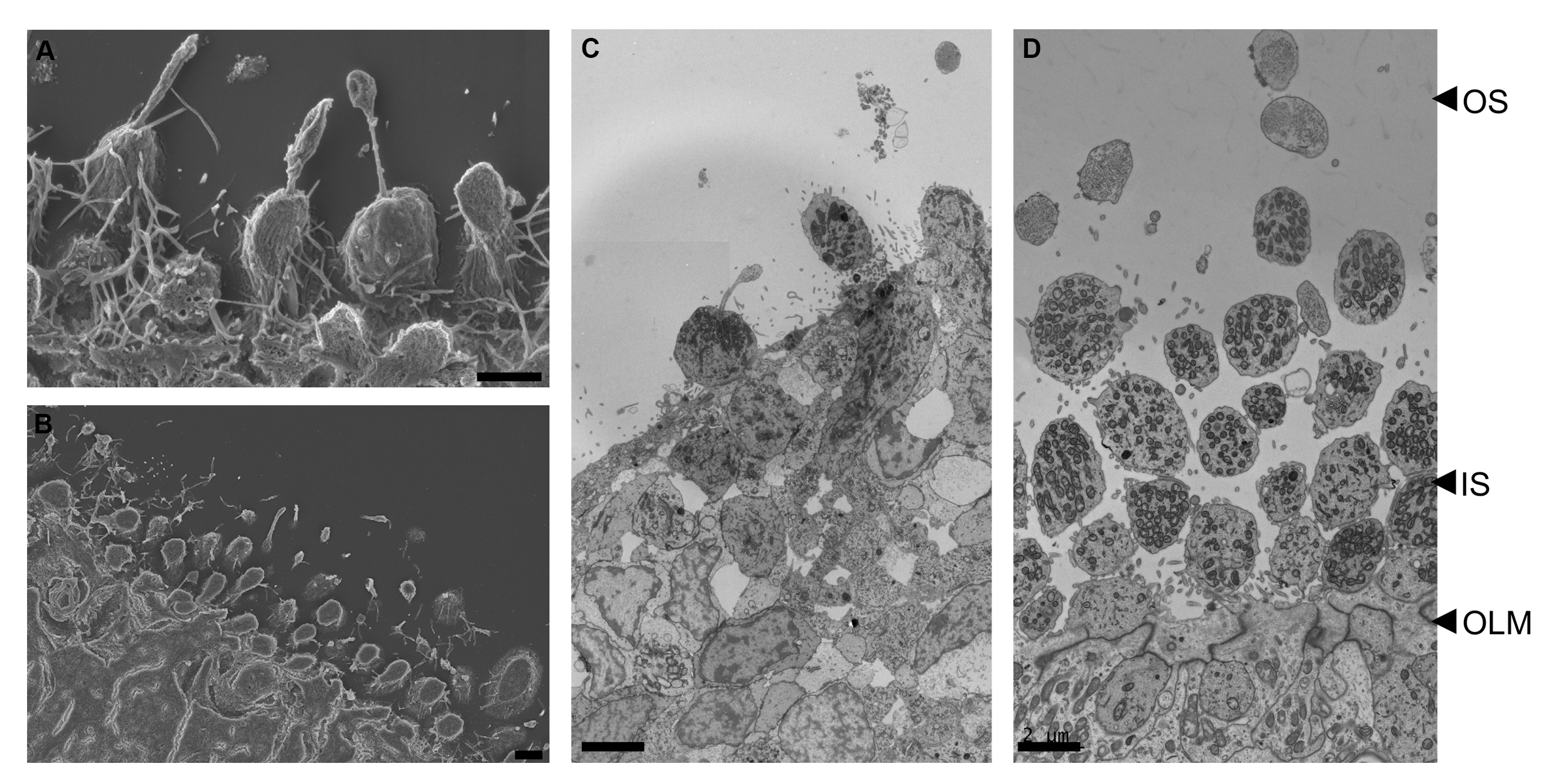


**Figure S5.**


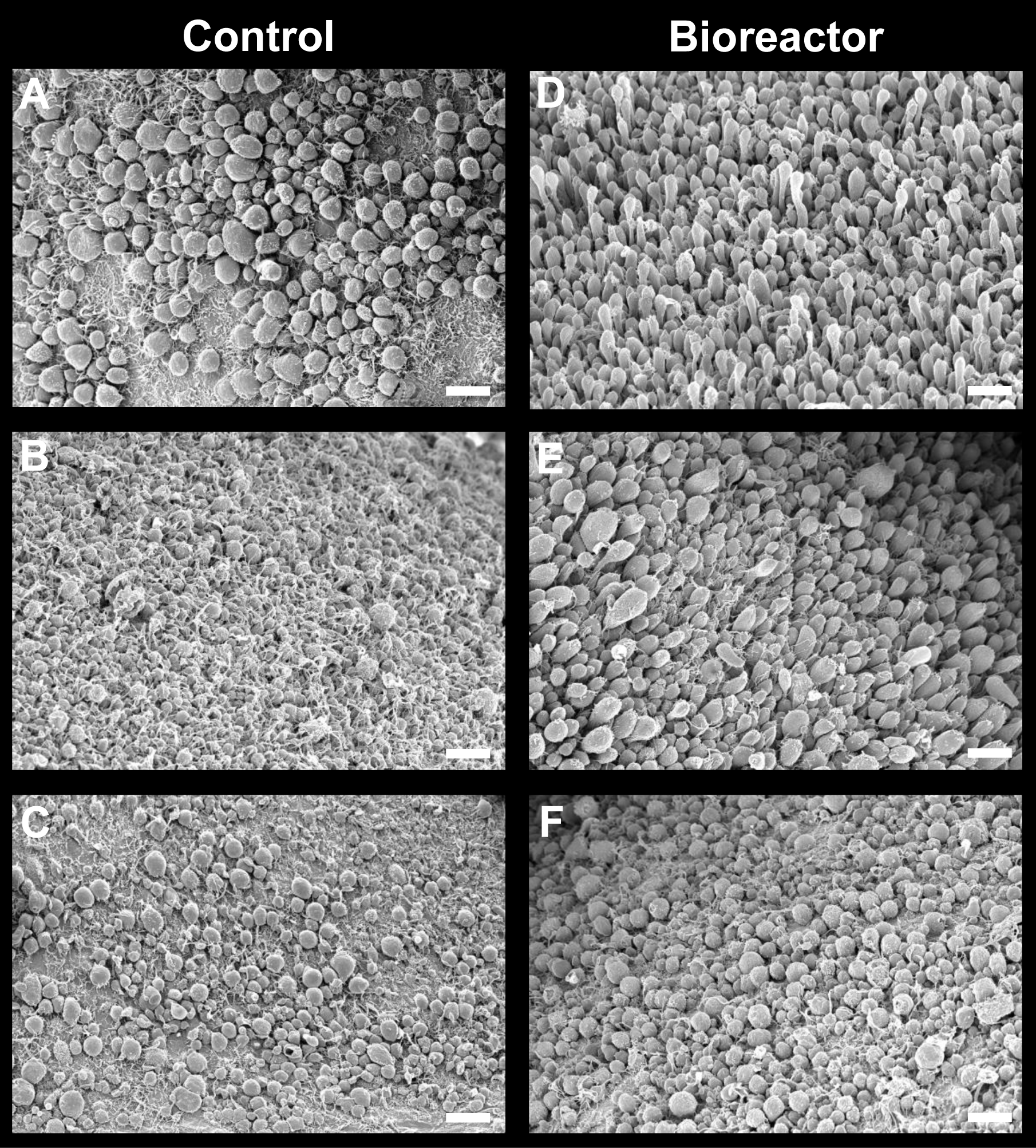


**Table S1. Antibodies used**

Supplement: Supplementary file 2 — Figure S1. Flow cytometric analysis and quantification of proportion of RECOVERIN/CD73 and CD133/CD73 double-positive cells within RECOVERIN and CD133 photoreceptor-positive populations. Representative FC plots of control vs bioreactor retinal organoids. A FC quantification of CD133/CD73 double-positive developing rods within CD133-positive population. B Quantification of RECOVERIN/CD73 double-positive mature photoreceptor cells by gating only in RECOVERIN-positive live cell population. Error bars, mean ± SEM; n = 50 retinal organoids, N = 3–4 independent differentiation experiments carried out per control or bioreactor condition; *P < 0.05, **P < 0.01, two-tail unpaired t test with Welch’s correction. Figure S2. Flow cytometry gating strategy employed for all flow cytometric analysis for each individual sample. A Dead cells excluded by using DRAQ7 vs FSC-A (or SYTOX Blue vs FSC-A; data not shown). Cellular aggregates gated out (FSC-A vs FSC-H) to ensure only single live cells (SSC-A vs FSC-A) used for subsequent analysis. B Representative plots of control vs bioreactor for RECOVERIN staining. Gates drawn using only secondary control samples for both control and bioreactor samples. C Representative plots of gating strategy used for CD73 staining in combination with CD133 antibody staining for both control and bioreactor samples. Unstained and fluorescence minus one (FMO) controls for CD73 and CD133 used to define positive fraction of cells for both control and bioreactor samples. D Representative plots for RECOVERIN and CD73 staining. Unstained and FMO gating controls used to determine RECOVERIN and CD73-positive cells for both control and bioreactor samples. Figure S3. Immunofluorescence analysis showing Müller glia (CRALBP-positive) and photoreceptor (RECOVERIN-positive) cells of week 15 retinal organoids in control (A) and bioreactor (B) conditions. Scale Bars: 200 μM. Figure S4. SEM and TEM images of hPSC-derived retinal organoid OLM regions. A, B SEM image show [file 13287_2018_907_MOESM1_ESM.docx]
